# Advances and Future Directions in Information Security and Data Privacy

**DOI:** 10.3390/e27101004

**Published:** 2025-09-26

**Authors:** Mina Alishahi

**Affiliations:** Department of Computer Science, Open University, 6401 DL Heerlen, The Netherlands; mina.sheikhalishahi@ou.nl

## 1. Introduction

In the last decade, information security and data privacy have emerged as defining concerns in the digital era, shaping technological innovation, policy discourse, and societal trust [1,2,3,4]. The increasing integration of networked systems into everyday life—from embedded devices in critical infrastructures to ubiquitous Internet of Things (IoT) applications—has led to unprecedented volumes of sensitive data being collected, processed, and transmitted [2,5,6,7,8]. While advances in cryptography, secure architectures, and privacy-preserving algorithms have brought substantial progress, the sophistication of adversarial attacks, the complexity of multi-layered systems, and the rise of AI-driven applications continue to challenge conventional approaches to security [2,5,6,9,10,11,12].

The research community has responded with a wide array of methods, spanning from low-level physical-layer protections to high-level policy and governance frameworks [7,11,13,14]. Yet, important questions remain: How can privacy be preserved without degrading functionality? How can systems be secured in environments with imperfect information or untrusted intermediaries? How should emerging AI technologies be integrated responsibly without exacerbating security vulnerabilities?

This Special Issue, *Information Security and Data Privacy*, captures these questions at a moment of rapid change. Across eight contributions—spanning embedded systems, IoT, AI-based privacy methods, federated learning, anonymization, wireless channel security, and next-generation architectures—the collection reflects both the maturity and the evolving frontiers of the field. The works presented here are united by a commitment to tackling real-world security and privacy challenges through rigorous, innovative, and often interdisciplinary approaches.

## 2. Overview of Published Articles

Two contributions address the reliability and integrity of software systems in mission-critical and resource-constrained environments. Zhao et al. (Contribution 1) present *ESfix*, an embedded program repair tool designed to effectively eliminate concurrency defects, with applications in aerospace, medical devices, and other domains requiring high assurance. Wu et al. (Contribution 3) propose a multi-factor authentication-based remote data backup scheme that strengthens security against data loss and tampering, even in untrusted backup environments.

Privacy and security in the IoT domain are tackled by Zhai et al. (Contribution 2), who develop a coercion-resistant attribute-based encryption scheme with policy revocation—an important step toward protecting sensitive data in large-scale IoT ecosystems. At the physical layer, Ta et al. (Contribution 7) introduce a novel noise injection strategy that guarantees zero secrecy outage probability under imperfect channel state information, offering robust protection in wireless communication scenarios.

The intersection of AI and privacy is represented in three works. You et al. (Contribution 4) leverage diffusion models to generate face privacy-protected images with high visual quality, addressing shortcomings of existing obfuscation methods. Li et al. (Contribution 5) propose LF3PFL, a local federalization-based federated learning framework that enhances privacy without sacrificing model performance. Khatir et al. (Contribution 6) present a greedy, information-theoretic clustering algorithm for anonymizing microdata, balancing privacy with data utility in k-anonymity contexts.

Finally, Kang et al. (Contribution 8) provides a broad perspective with a comprehensive survey on zero trust security, synthesizing theoretical foundations, implementation strategies, and the paradigm’s implications for future cybersecurity architectures.

## 3. Conclusions and Future Directions

Taken together, these contributions advance the state of the art in multiple dimensions: securing embedded systems, safeguarding IoT communications, fortifying wireless channels, anonymizing data, and enhancing privacy in AI-driven and collaborative learning environments. The diversity of methods—ranging from algorithmic innovations to architectural paradigms—underscores the multifaceted nature of modern security challenges.

Looking forward, the field will benefit from:

- **Integrated approaches** that combine complementary techniques, such as merging zero trust principles with privacy-preserving machine learning.
- **Scalable, adaptive defenses** capable of responding in real time to evolving threats, especially in IoT and federated environments.
- **Human-centered security** that accounts for usability, policy, and governance alongside technical robustness.
- **Bridging theory and practice**, ensuring that cutting-edge methods can be deployed at scale without prohibitive costs or complexity.

This Special Issue not only reflects current achievements but also lays out a roadmap for future research, inviting continued cross-disciplinary collaboration to address the complex and dynamic landscape of information security and data privacy.
